# Gastrointestinal parasites of *Leontopithecus chrysomelas* in the Atlantic Forest, Brazil

**DOI:** 10.1590/S1984-29612022005

**Published:** 2022-02-21

**Authors:** Lilian Silva Catenacci, Janilda Barros Santiago Oliveira, Kristel Myriam De Vleeschouwer, Leonardo de Carvalho Oliveira, Sharon Lynn Deem, Severino Cavalcante de Sousa, Karina Rodrigues dos Santos

**Affiliations:** 1 Programa de Pós-Graduação em Tecnologias em Animais de Interesse Regional – PPGTAIR, Universidade Federal do Piauí, Teresina, PI, Brasil; 2 Programa de Pós-Graduação em Saúde Animal na Amazônia – PPGSAAM, Universidade Federal do Pará, Castanhal, PA, Brasil; 3 Centre for Research and Conservation, Royal Zoological Society of Antwerp, Antwerp, Belgium; 4 Saint Louis Zoo Institute for Conservation Medicine, Saint Louis, MO, United States; 5 Departamento de Medicina Veterinária, Universidade Federal do Piauí, Teresina, PI, Brasil; 6 Departamento de Ciência, Universidade Estadual do Rio de Janeiro, Rio de Janeiro, RJ, Brasil; 7 Bicho do Mato Instituto de Pesquisa, Belo Horizonte, MG, Brasil; 8 Universidade Federal do Delta do Parnaíba, Parnaíba, PI, Brasil

**Keywords:** Lion-tamarin, new world monkeys, cabruca, coproparasitological methods, helminths, protozoans, Mico-leão, primatas neotropicais, cabruca, métodos coproparasitológicos, helmintos, protozoários

## Abstract

We performed coproparasitological testing of free-living golden-headed lion tamarins, *Leontopithecus chrysomelas,* using the Hoffmann-Pons-Janner method. In total, we collected 118 samples from ten groups: four living in Federal Protected Area and six living in Non-Protected Areas of cocoa farms. Eggs from parasites of the Acanthocephala phylum and Spiruridae, Ancylostomatidae, Ascarididae and Oxyuridae families were identified, as well as the genus *Strongyloides* (Nematode: Strongyloididae) and phylum Apicomplexa. This is the first description of infection with coccidian, Trichuridae family and *Strongyloides* spp. in *L. chrysomelas*. A total of 48% (n= 57) of the animals were infected and the highest prevalence (37.2±SD 8.72, n = 44) was for Acanthocephalidae, followed by Spiruridae (8.5±SD 5.03, n = 10). There was no difference in parasite prevalence by age classes or sex. However, we found higher diversity and prevalence of parasites in animals living in the Federal Protected Area. These results suggest that intestinal parasites may be influenced by environmental factors, such as the management of the areas where the animals live, in addition to the feeding behavior of *L. chrysomelas* and distinct transmission strategies of parasites. The combination of ecological and demographic data combined with parasitological studies may contribute to conservation programs for this species.

## Resumo

Foram realizados testes coproparasitológicos de micos-leões-dourados de vida livre, *Leontopithecus chrysomelas*, usando-se o método de Hoffmann-Pons-Janner. No total, foram coletadas 118 amostras de dez grupos: quatro grupos residentes em Área de Conservação Federal e seis grupos em Área não protegida de fazendas de cacau. Ovos de parasitas do filo Acantocephala e das famílias Spiruridae, Ancylostomatidae, Ascarididae, Oxyuridae foram identificados, bem como o gênero *Strongyloides* (Nematoda: Strongyloididae) e o filo Apicomplexa. Esta é a primeira descrição de infecção de coccídeos, família Trichuridae e *Strongyloides* spp. em *L. chrysomelas*. Um total de 48% (n = 57) dos animais estavam parasitados e a maior prevalência (37,2 ±DP 8,72, n = 44) foi para Acanthocephalan, seguido por Spiruridae (8,5±DP 5,03, n = 10). Não houve diferença na prevalência do táxon de parasita por idade ou sexo. No entanto, foi encontrada maior diversidade e prevalência de parasitas em animais que vivem na Unidade de Conservação Federal. Esses resultados sugerem que os parasitas intestinais podem ser influenciados por fatores ambientais, como o manejo das áreas, além do comportamento alimentar de *L. chrysomelas* e distintas estratégias de transmissão dos parasitas. A combinação de dados ecológicos e demográficos com estudos parasitológicos podem contribuir para programas de conservação dessa espécie.

## Introduction

Environmental changes and ecological disturbances caused by both anthropogenic and natural causes have been shown to influence parasitic diseases in a number of species (Bongers & Ferris, 1999; Patz et al., 2000; Cleaveland et al., 2001). These disturbances can alter the ecological balance between the vector, host, and parasite, which may impact the epidemiology of parasitic diseases (Daszak et al., 2000; Patz et al., 2000; Altizer et al., 2003; Molina et al., 2019). Parasitic infections have been identified as a critical component to be considered in conservation biology (Daszak et al., 2000; Altizer et al., 2003) because the impact of parasitic infections in free-living populations may affect the density and distribution of host species (Cleaveland et al., 2001; Nunn et al., 2003, 2004; Solórzano-García & Pérez-Ponce de León, 2017).

Primates are particularly vulnerable to the effects of parasites due to their social behavior, such as cohesive social group living, which facilitates parasite transmission (Altizer et al., 2003; Costa et al., 2020). In addition, several species of primates are omnivorous and eat invertebrates, which increases the likelihood of trophic transmission (Nunn et al., 2003; Pedersen et al., 2005; Oliveira et al., 2017). There is a wide diversity of organisms that parasitize non-human primates (Solórzano-García & Pérez-Ponce de León, 2018); which are adapted to their hosts and thus, cause few pathological issues. However, others have been linked to significant and even fatal damage, such as helminths of the Acanthocephala phylum (Chandler 1953; Pissinatti et al., 2007; Catenacci et al., 2016a; Oliveira et al., 2017). There remains a paucity of data on intestinal parasite prevalence and diversity for Brazilian endangered primate species, including the Brazilian species of tamarins (Monteiro et al., 2007a; Stoner, 1996; De Vleeschouwer et al., 2011; Solórzano-García & Pérez-Ponce de León, 2018). Lion tamarins (Callitrichidae: *Leontopithecus* spp.) are small arboreal primates (weighing between 586 g and 653 g) (Oliveira et al., 2011) which live in small social groups (Raboy et al., 2004) of an average of seven individuals per group. *Leontopithecus chrysomelas* are endemic to the southern Atlantic Forest of Bahia, Brazil (Kierulff et al., 2002; Rylands, 1993; De Vleeschouwer et al., 2011) where they live in a highly fragmented area (Guy et al., 2016).

We conducted a field survey in order: (1) to describe and compare gastrointestinal parasites found in *L. chrysomelas* groups, (2) to analyze the prevalence of helminths found in the lion tamarins with respect to age and sex classes and geographic distribution, and (3) to determine ecological and epidemiological factors associated with the relationship between hosts and parasites in different landscapes in the Atlantic Forest, southern Bahia, Brazil. We hypothesized that the highest abundance of parasites would be found in groups that lived in non-protected areas, while the highest prevalence would be found in groups that inhabit pristine forests (“protected areas”).

## Material and Methods

### Area and study groups

**Figure 1:**
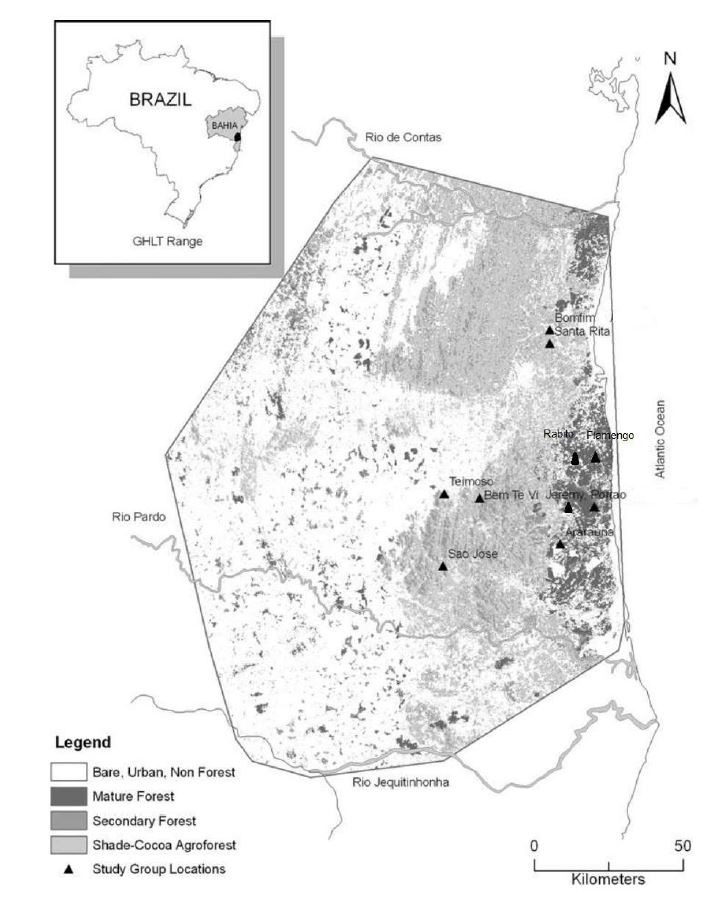
Geographical distribution of golden-headed lion tamarin the southeast of Bahia, Brazil and location of the study sites.

The study was conducted in areas belonging to the Bahia Atlantic Forest domain, Brazil. Across this region, there are differences in land use and anthropogenic pressures. We collected data from ten groups of *L. chrysomelas*; four groups in a Federal Protected Area (Una Biological Reserve-REBIO) and six in a Non Protected Areas of cocoa farms (called Cabruca) and fragments of forest remains in cocoa farms (Figure 1).

Una Biological Reserve (REBIO-Una) is the largest fully protected Federal unit in the region, comprising 18,515 hectares covered by continuous Atlantic Rainforest: the interior parts of the reserve are largely tall forest but other areas are in different stages of regeneration following disturbances prior to the reserve’s establishment. The unprotected areas in this study belong to fragments of forest remains in cocoa farms and cabruca, which is an agroforest system defined as a dynamic and ecologically-based natural resource management practice where growing trees of cocoa are shaded by native trees (Oliveira et al., 2011). The unprotected areas present specific challenges to groups of lion tamarins, such as lower resource availability (i.e., sleeping trees), higher exposure to predators, higher hunting levels and human contact in comparison with groups living in the protected area (Raboy & Dietz, 2004; De Vleeschouwer et al., 2011; Oliveira et al., 2017).

Based on previous studies, we know that the three groups of golden-headed lion tamarins living in REBIO have an average size of 4.67 individuals per group, average density of 0.059 individuals per hectare, and average living area of 84.9 hectares (De Vleeschouwer et al., 2011; Catenacci et al., 2016b). For the seven groups inhabiting farms outside the conservation unit, the average size of the groups was 7.2 individuals per group, average density of 0.15 individuals per hectare, and average living area of 54.6 hectares (Oliveira et al., 2011).

### Capture, chemical restraint and sample collection

The study was conducted from February 2008 to July 2010 as part of long-term ecological and health monitoring; and involved examination of feces collected from 118 lion tamarins (*L. chrysomelas*): 76 males and 42 females, with age classes of 18 juveniles, 23 subadults, and 77 adults (Table 1). Primates were captured individually using Tomahawk live traps (Rosa minas®) (48.3 x 15.2 x 15.2 cm) baited with bananas and placed on platforms 1.5m above ground in areas used by tamarin groups (Dietz et al., 1996). Once captured, all the animals were taken to a field laboratory for processing, and then released the following day at the same location where they were captured. All water and food was removed at 4 hr prior to anesthesia. Individual tamarins in traps were anaesthetised by hand injection of ketamine hydrochloride (10 mg/kg; i.m.) and midazolam hydrochloride (0.3 mg/kg; i.m.) in a single syringe using a 22 g needle. During the chemical restraint, a physical examination was performed and biological samples (feces) were collected from each animal. Most of the animals defecated during the physical and chemical restraint and the feces had to be collected from newspaper on the bottom of the trap. Feces were collected immediately after the defecation and the newspaper was replaced and the floor cleaned with sodium hypochlorite between each animal procedure to avoid contamination. In a few animals we had samples taken directly from the rectum. The following data were collected for each animal: sex, age group, animal identification and body weight, according to Dietz et al. (1996). All procedures were performed by veterinarians and biologists, using personal protective equipment (e.g., disposable gloves, masks and coat).

**Table 1:** Characterization of golden-headed lion tamarin groups, according to sex, age and number of fecal samples collected in southern Bahia, Brazil.

	**Study site**	**Number of groups**	**Sex**	**Age range** *
**Protected Area**	REBIO-UNA	4	22 F	14 J
			42 M	16 SA
				34 A
**Non- Protected Area**	Mosaic	6	20 F	4 J
			34 M	7 SA
				43 A
**Total of samples collected (one feces/animal)**		10	42 F	18J
			76M	23SA
				77A

* F: Female; M: Male; J: Juvenile; SA: subadult; A: adult.

### Parasitological exams

Fecal samples weighed 1.0 to 3.0 g. After weighing, each sample was immediately preserved in 4% buffered formalin solution and transported at room temperature later parasitological analysis (Monteiro et al., 2007a). Preserved samples were examined for presence/absence of parasite eggs under direct light microscopy (10x, 40x, 100x) using a sedimentation test, Hoffmann-Pons-Janer (Katagiri & Oliveira-Sequeira, 2007). The identification of the parasites eggs was based mainly on morphology, in comparison with previously described studies (Vicente et al., 1997; Monteiro et al., 2003; Tavela et al., 2013; Catenacci et al., 2016a; Oliveira et al., 2017).

Unfortunately, some intestinal parasites have similar egg morphologies within the same genus (Brandão et al., 2009; Sales et al., 2010), which prevented the identification of these to the genus or species levels.

### Statistical analysis

Prevalence and the confidence intervals (95%) were calculated as the total infected individuals divided by the total individuals sampled. The percentage of infected hosts was estimated for each parasite taxa; in addition, we also quantified the number of hosts that were infected by at least one helminth species. To determine whether there was a difference in parasitic infections (richness and prevalence) based on sex, age group and habitat of each animal, we used a chi-square test with confidence level of <0.05. All the tests were performed using the statistical analysis system - SAS, 9.1 (SAS, 2003).

### Ethical note

Housing conditions, exploration testing, and isolation testing met with protocols approved by the appropriate institutional animal care committee (Ethics Committee on Animal Experimentation at the Universidade Estadual de Santa Cruz, number13/07). The captures were also authorized by the Brazilian Environmental Agency (IBAMA/ICMBio) permit numbers 12334-1, 18444-1, 113/2007 and 15025/2009. The authors declare that they have no conflict of interest.

## Results

Forty-eight percent of the golden-headed lion tamarins (N= 57) were found parasitized, and 38% (n=45) of them had at least one family of helminth or Apicomplexa phylum and 10% (n=12) showed more than one parasite in the same fecal samples (Table 2).

**Table 2:** Prevalence of helminth eggs according to the sex of subjects from golden-headed- lion tamarins captured in the wild in southern Bahia, Brazil.

**Prevalence**	**Sex**		
	**Male** P±ME(N)*	**Female** P±MEI(N)	**Total** P±ME(N)
**Absent of parasite**	34.7±8.59 (41)	16.9±6.76 (20)	51.6±9.02 (61)
**Only 1 parasite**	22±7.47 (26)	16.1±6.63 (19)	38.1± 8.76(45)
**2 or more parasites**	7.6±4.78 (9)	2.5±2.82 (3)	10.1±5.44(12)
**Total**	64.3±8.64(76)	35.5±8.63(42)	100 (118)

* P= prevalence; ME: margin of error; N=number of samples.

^A and B in the same rows means statistical significance.^

**Figure 2:**
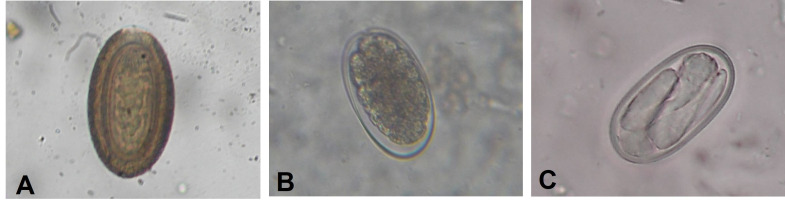
Eggs of the Acanthocephalan and the nematode Ancylostomidae, Strongyloididae families and Apicomplexa phylum: A) egg of the Acanthocephalan; B) Ancylostomatidae; C) Strongyloididae.

Considering richness, we detected eight different parasite taxa: Ancylostomatidae, Ascarididae, Oxyuridae, Spiruridae and Trichuridae families, one from the genus *Strongyloides*, one from Apicomplexa phylum and one for Acanthocephala phylum (Table 3; Figure 2).

**Table 3:** Overall prevalence and frequency of helminth eggs in stool of Golden-headed lion tamarins in a Protected area (REBIO-Una) and unprotected areas in Southern Bahia, Brazil.

**Area**	**Total N**	**Overall prevalence P±ME (N)** **	**Frequency of helminth eggs (N)** *							
			**Acan**	**Spi**	**Oxy**	**Anc**	**Asc**	**Str**	**Tri**	**Coc**
Protected area	61	63.8± 8.67 (39)	84.6(33)	33.3 (7)	2.5(1)	5.1(2)	7.7 (3)	2.5 (1)	2.5 (1)	2.5 (1)
Unprotected areas	57	31.6± 8.39 (18)	61.1 (11)	16.6(3)	11.1(2)	5.5(1)	0	0	0	0
**Both Areas**	118	48.3±9.02 (118)	37.2 (44)	8.5(10)	2.5(3)	2.5(3)	2.5(3)	0.9(1)	0.9(1)	0.9(1)

* Acanthocephalidae (Acan), Spiruridae (Spi), Oxyuridae (Oxy), Ancylostomatidae (Anc), Ascarididae (Asc), *Strongyloides* spp., Coccidian, Trichuridae (Tri). Values are percentages related to each area (nominal data are in brackets).

** P= prevalence; ME: margin of error; N=number of samples. The frequencies of helminth eggs were calculated considering only the positive samples.

The Acanthocephala and Spiruridae family had higher frequency among the positive samples (77.2± 7.57 and 17.5± 6.86, respectively) and were found in all study areas. The groups living within the Federal Protected Area had more samples with parasites and presented more diversity of parasites than groups from Non-Protected Areas (p=0,0385). Eggs from the Ascarididae and Trichuridae families, genus *Strongyloides* spp. (Strongyloididae) and coccidian had lower prevalence and were present only at the Federal Protected Area (Table 3 and Table 4). Despite the parasitism, all the tamarins seemed to be in good physical condition and no sex (p=0.692) or age effect (p=0.0534) were found (Table 5).

**Table 4:** Prevalence of helminth eggs according to age groups of specimens from Golden-headed tamarins captured in the wild in southern Bahia, Brazil.

Prevalence	Age Group P±ME(N)*			**Total**
	**juvenile**	**Subadult**	**Adult**	
**Absent of parasites**	5.1±3.97 (6)	8.5±5.03 (10)	38.1±8.76 (45)	51.6±9.02 (61)
**only 1 parasite**	9.3±5.24 (11)	7.6±4.78 (9)	21.2±7.37 (25)	38.2±8.76 (45)
**only 2 parasites**	0.8±1.8 (1)	3.4±3.27 (4)	7±4.25 (5.9)	11.3±5.6 (12)
**Total**	15.2±6.48(18)	19.5±7.15(23)	66.3±8.53 (77)	100 (118)

* Prevalence; ME: margin of error; N=number of samples.

**Table 5:** Statistical results of golden-headed lion tamarin groups, according to taxon of parasite, sex and age of fecal samples collected in southern Bahia, Brazil.

**Variables**	**Study site**		**P value**
	**Protected Area**	**Non- Protected Area**	
**Taxon of parasite**	1.015^A^	1.185^B^	0.0385
**Sex**	1.340^A^	1.373^A^	0.692
**Age**	1.71^A^	1.45^A^	0.0534

## Discussion

This is the first description of infection with the Coccidian, Trichuridae family and the genus *Strongyloides* spp. in *L. chrysomelas*. Similar to other studies of free-living groups of *Leontopithecus* spp. and *Callithrix* spp., the acanthocephalan were the parasites with highest prevalence (Costa et al., 2020; Monteiro et al., 2003, 2007a, 2010; Sales et al., 2010). While we found a prevalence of 37.2± SD 8.72 (N=44) of Acanthocephalan, a total of 33% (N=8) of the samples collected by Costa et al. (2020) had feces with parasites and all of them registered the presence of Acanthocephala. From the study of Monteiro et al. (2010), other groups of *L. chrysomelas* had almost 80% (N=68) positive samples and 49% presented with Acanthocephalan eggs. The Spiruridae family, as observed in the present study, seems to be the second most prevalent eggs found among the *L. rosalia* and *L. chrysomelas* groups with prevalence described from 24% (n=199) to 38% (N=68) (Monteiro et al., 2003; 2007b; 2010). Considering the other parasites, Monteiro et al. (2003; 2007a; 2010) also found a low prevalence of Ascarididae, Oxyuridae and Ancylostomatidae families in free-living groups of *L. rosalia* and *L*. *chrysomelas.*

The feeding behavior (Tavela et al., 2013) of the lion tamarins and the distinct transmission strategies of Acanthocephalan and Spiruridae, in contrast with the other intestinal parasites reported, may explain the different prevalence among them. As *L. chrysomelas* are frugivorous-insectivorous (Kierulff et al., 2002; De Vleeschouwer et al., 2011; Catenacci et al., 2016b), they feed on arthropods (cicadas, grasshoppers and cockroaches), which may serve as intermediate hosts, with infective acanthellae and L3, respectively, which need to be ingested to develop the next step of their life cycles . The primate definitive host gets infected by eating cockroaches and beetles (e.g., Blattodea and Coleoptera), containing third-stage larvae of these parasites (Chandler, 1953; Travassos et al., 1969; Urquhart et al., 1998; Weber & Junge, 2000; Pedersen et al., 2005). Transmission between lion tamarins also may occur by sharing contaminated food (invertebrates), a behavior commonly observed for this species, or sites of food found in bromeliads, increasing chances of infection among more sociable individuals (Costa et al., 2020).

With respect to other parasites found, transmission might occur via fomites or contaminated water and soil (Bongers & Ferris, 1999). Since these primates are predominantly arboreal (Rylands, 1993; Raboy & Dietz, 2004) a lower incidence was expected.

As found for *Callithrix* sp. (Sales et al., 2010), no difference in prevalence based on sex and age classes was registered, probably because the entire social group eat invertebrates, especially insects (Catenacci et al., 2016b; Oliveira et al., 2011).

The higher biodiversity of parasites registered in a Federal Protected area (REBIO) suggests that high richness in conserved tropical forests may extend to parasite diversity as well (Monteiro et al., 2007a), as we expected considering the dilution effect (Catenacci et al., 2021).

Eggs from Ascarididae, Trichridae, Strongyloididae and coccidian oocysts were reported only in groups from inside the natural reserve. Human disturbed environments, such as cabruca and small patches of disconnected forest, tend to have lower biodiversity than REBIO (Al-Shorbaji et al., 2016; Guy et al., 2016; Costa et al., 2020). Furthermore, the management differences of the environment are essential to determine the establishment and reproduction of the parasites (Grundmann et al., 1976; Bongers & Ferris, 1999; Nunn et al., 2003). Inside continuous forest, such as the REBIO, moderate temperatures, high humidity and litter tends to form a microclimate that contributes to the reproduction of invertebrates and the maintenance of most parasites which are transmitted through soil and water (Bongers & Ferris, 1999; Patz et al., 2000; Urquhart et al., 1998). However, in areas outside the conservation units, the management of trees and associated soil and litter (Oliveira et al., 2011), deforestation with areas without trees and thus more exposure to sun favors an increase of temperature in the region and decrease in humidity in these places, thus disfavoring the parasite cycle and leading to a reduction in the diversity of helminths that can infect vertebrate hosts, such as lion tamarins (Costa et al., 2020).

It is important to emphasize that high diverse parasite community may not be evidence of greater impacts of parasites on host, or at least should not be assumed to be a negative quality from an ecological perspective. However, parasitological studies can shed light on host health status and vulnerability to parasitic infections in threatened species such as the *L. chrysomelas*. From the point of view of conservation, the finding of Acanthocephalan eggs may represent a risk for populations of golden-headed lion tamarins in the wild that already may face stress factors such as predation, hunting and human contact (Raboy & Dietz, 2004; Guy et al., 2016; Oliveira et al., 2011). Monteiro et al. (2010) also stated that Acanthocephalan infection results in a significant reduction in tamarin health, which can potentially lead to their death. Additionally, Acanthocephalan infection is described in the literature as one of the most severe helminthiasis, characterized by bleeding and convulsions, with infected animals often having anorexia, weight loss, anemia, septicemia leading to death (Weber & Junge, 2000; Pissinatti et al., 2007; Catenacci et al., 2016a; Oliveira et al., 2017). Despite the wide distribution of acanthocephalan in free-living populations, when compared to captive species, it appears to be less harmful (Soto-Calderón et al., 2016), often being an accidental finding during necropsy (Oliveira et al., 2017). As described by Costa et al. (2020), the lack of other health concerns of free-ranging individuals of golden-headed lion tamarins infected by the acanthocephalan suggests that animals might have developed an individual tolerance to parasites. However, future studies are necessary to confirm this hypothesis. It must be noted, in conservation strategies that involve the movement of animals, gastrointestinal parasites may become more harmful in animals stressed from movements and potentially immunocompromised. We recommend that reintroduction and translocation programs previously developed for *L. chrysomelas* in other areas (Molina et al., 2019; Santos et al., 2019), and the establishment of biological corridors in Southern Atlantic Forest proposed by Dosen et al. (2017), must consider parasite transmission dynamics.

## Conclusion

In this study we demonstrate differences in the richness of parasites between the *Leontopithecus chrysomelas* groups based on the environment where they live, and that these data increase our ecological knowledge of the species, especially their relationship with Acantocephalan and Spiruridae parasites. The development of integrated research that includes the eco-epidemiology of parasites communities is important for primate conservation.
